# An Investigation of a (Vinylbenzyl) Trimethylammonium and *N*-Vinylimidazole-Substituted Poly (Vinylidene Fluoride-Co-Hexafluoropropylene) Copolymer as an Anion-Exchange Membrane in a Lignin-Oxidising Electrolyser

**DOI:** 10.3390/membranes11060425

**Published:** 2021-06-02

**Authors:** Patrick J. McHugh, Arindam K. Das, Alexander G. Wallace, Vaibhav Kulshrestha, Vinod K. Shahi, Mark D. Symes

**Affiliations:** 1 WestCHEM, School of Chemistry, University of Glasgow, University Avenue, Glasgow G12 8QQ, UK; p.mchugh.1@research.gla.ac.uk (P.J.M.); arindamkumar91@gmail.com (A.K.D.); A.G.Wallace@soton.ac.uk (A.G.W.); 2 Central Salt and Marine Chemicals Research Institute, Bhavnagar 364002, Gujarat, India; vaibhavk@csmcri.res.in

**Keywords:** anion-exchange membrane, lignin oxidation, electrolyser, fluoropolymer

## Abstract

Electrolysis is seen as a promising route for the production of hydrogen from water, as part of a move to a wider “hydrogen economy”. The electro-oxidation of renewable feedstocks offers an alternative anode couple to the (high-overpotential) electrochemical oxygen evolution reaction for developing low-voltage electrolysers. Meanwhile, the exploration of new membrane materials is also important in order to try and reduce the capital costs of electrolysers. In this work, we synthesise and characterise a previously unreported anion-exchange membrane consisting of a fluorinated polymer backbone grafted with imidazole and trimethylammonium units as the ion-conducting moieties. We then investigate the use of this membrane in a lignin-oxidising electrolyser. The new membrane performs comparably to a commercially-available anion-exchange membrane (Fumapem) for this purpose over short timescales (delivering current densities of 4.4 mA cm^−2^ for lignin oxidation at a cell potential of 1.2 V at 70 °C during linear sweep voltammetry), but membrane durability was found to be a significant issue over extended testing durations. This work therefore suggests that membranes of the sort described herein might be usefully employed for lignin electrolysis applications if their robustness can be improved.

## 1. Introduction

Due to the finite supply of fossil fuels and the well-understood relationship between their widespread usage and negative effects on the global climate, there is major interest in the development and implementation of energy solutions which do not result in greenhouse gas emissions [1]. Renewables such as wind, solar and tidal all have great potential in this regard, but are limited by the fact that they are fluctuating, intermittent sources of energy. For such renewable power sources to meet global energy demand and become long-term solutions to the current energy crisis, we therefore require a suitable means of storing the energy as and when it is available [2,3]. On this basis, the use of renewables to produce the fuel hydrogen (H_2_) by electrolysis of water has long been considered as one of the most promising means of energy storage, as hydrogen has many attractive qualities as a fuel [4]. At the time of writing, the majority of industrial-scale water electrolysis is performed using a corrosive liquid alkaline electrolyte, with an asbestos diaphragm which separates the anodic and cathodic chambers and prevents the product gases from mixing (which would otherwise form a highly explosive mixture) [5]. Although this liquid alkaline electrolyte approach is relatively inexpensive in terms of capital costs, the maximum operational current densities are limited, and the pressure in each chamber must be well managed to prevent gas cross-mixing via permeation across the membrane [6,7,8]. The operational costs of liquid alkaline electrolyte cells are therefore sub-optimal.

The use of electrolysers employing solid polymer electrolytes has emerged steadily over recent decades as a route by which some of the drawbacks of liquid-phase alkaline electrolyte cells can be overcome. For example, caustic electrolytes are no longer required, many of the membranes commonly used in such cells can withstand high pressure differentials without significant gas cross-mixing occurring, and much higher operational current densities can be achieved [9]. Proton-exchange membranes (e.g., Nafion) have been the subject of a large number of publications in this regard, and current densities >2 A cm^−2^ can be achieved [5,10], while the crossover rates of the gaseous products are kept low (although gas crossover is never entirely eliminated) [8,11]. In contrast to the liquid alkaline cells currently used in most large-scale industrial applications (which employ non-noble catalysts such as Ni and Co), proton-exchange membrane electrolysers require expensive noble metal catalysts and acid-resistant components. This is due to the harshly acidic environment generated at the electrode during electrolysis, and has financial implications which could impede the adoption of this technology on a commercial scale [8]. In this regard, the development of anion-exchange membrane electrolysers could be transformational, as such systems have the potential to work with non-noble metal catalysts, and the membranes themselves are often cheaper to produce than Nafion [12,13]. Notwithstanding recent reports of excellent conductivity and stability [14,15,16,17], the conductivity of OH^−^ ions is generally lower in anion-exchange membranes than is the proton conductivity in proton-exchange membranes; however, in a practical setting, the higher operational costs of such anion-exchange membrane electrolysers might well be offset by their (probably) lower capital costs.

Electrolytic water splitting can be expressed in terms of its two constituent half reactions: the oxygen evolution reaction (OER), and hydrogen evolution reaction (HER). Under standard conditions, the thermodynamic minimum voltage required to electrolyse water is 1.23 V. In practice, additional voltages are required to carry out the HER and OER at appreciable currents, on account of energy barriers related to concentration, ohmic resistances, and the kinetics of each half reaction [18]. In water electrolysis, the primary source of these additional voltages, called overpotentials, is the OER, owing to the kinetic demands of carrying out the four-electron, four-proton production of O_2_ [19]. Although this energy requirement can be lowered by employing appropriate electrocatalysts, an intriguing alternative to this is to replace the OER with an anode couple that does not (at least in theory) require such significant overpotentials. To this end, the electrolysis of organic compounds as a route to H_2_ production has garnered some interest [20,21,22,23,24]. Provided that the organic substrates that are being oxidised are renewable (e.g., they are derived from plant-based material), then such a system would allow the production of hydrogen from water at lower potentials than the direct electrolysis of water to O_2_ and H_2_ without adding to the long-term concentration of CO_2_ in the atmosphere. Ideally, candidates for these alternative renewable anodic feedstocks should also be plentiful, non-toxic and otherwise not on the pathway to other critical resources such as foodstuffs. Lignin, a highly aromatic, naturally-occurring polymer found in wood, fits this specification well. It is produced as a low-value side product of the Kraft pulping process in volumes of 40–50 million tonnes per year, making it the second most abundant source of renewable carbon and readily available at a low cost [25]. Using lignin as a substrate for the production of hydrogen from water could thus leverage value from this by-product, and indeed, the use of lignin in this capacity has been the subject of several publications [19,26,27,28,29,30,31,32,33,34]. However, the use of anion-exchange membranes in such lignin-converting electrolysers remains underexplored, with only a very few examples of such studies reported to date [30,35,36].

The research reported herein describes the synthesis of a novel anion-exchange membrane and the testing of its suitability for use in an anion-exchange membrane electrolyser for direct lignin electrolysis. The membrane in question (hereafter called “PVIB”) is a co-polymer of dehydrofluorinated poly (vinylidene fluoride-co-hexafluoropropylene) with (vinylbenzyl)trimethylammonium chloride and *N*-vinylimidazole. The performance of this membrane for lignin electrolysis was compared under a range of conditions to that of the commercially-available anion-exchange membrane Fumapem FAA-3-50, which (to the best of our knowledge) is the only anion-exchange membrane yet explored as a separator in a zero-gap lignin-oxidising electrolyser. Fumapem FAA-3-50 is cross-linked, generally non-reinforced anion-exchange membrane with a polyaromatic hydrocarbon backbone and utilising quaternary ammonium moieties to facilitate anion conduction [37]. PVIB therefore differs from Fumapem FAA-3-50 both in terms of the nature of its backbone (which is partly fluorinated) and in the incorporation of secondary imidazolium cationic units (see below). PVIB (whose synthesis has not been reported before), therefore seemed to us to offer some potential for increased conductivity and/or chemical stability compared to Fumapem FAA-3-50 when used as the anion-exchange membrane separator in a zero-gap lignin-oxidising electrolyser. With this in mind, we set out to test the performance of PVIB compared to that of Fumapem FAA-3-50 in an anion-exchange membrane lignin-oxidising electrolyser. It was found that the PVIB-based electrolyser performed comparably to an electrolyser using Fumapem for lignin oxidation over short timescales (with an applied potential of 1.2 V driving a current density of 4.4 mA cm^−2^ at 70 °C during linear sweep voltammetry), but that membrane durability was an issue over extended testing durations. Together, our results show that PVIB-based membranes could show promise for such electrolysis applications, if their robustness can be improved.

## 2. Experimental

### 2.1. Materials

Ultrapure deionised water (18.2 MΩ·cm) obtained from a Sartorius Arium Comfort combined water system was used in all experiments. Alkali (Kraft) lignin and sodium hydroxide (≥98%) were purchased from Sigma Aldrich and Honeywell, respectively. The electrodes used were commercial products purchased from FuelCellStore. The anode used was Pt/Ru catalyst (2 mg cm^−2^, 50% Pt/50% Ru wt/wt) impregnated on carbon cloth (410 μm thick microporous layer). The cathode used was a Pt/C catalyst (0.2 mg cm^−2^, 20% wt Pt) impregnated on identical carbon cloth to the anode. The commercial anion-exchange membrane FUMAPEM FAA-3-50 was purchased from FuelCellStore. Poly (vinylidene fluoride-co-hexafluoropropylene) (M_n_~130,000 g/mol), *N*-vinylimidazole and (vinylbenzyl) trimethylammonium chloride monomers were purchased from Sigma Aldrich and used without further purification. Dimethylacetamide and isopropyl alcohol were supplied by SD Fine-Chem Ltd. (Mumbai, India).

### 2.2. Flow Cell Components

**Figure 1:**
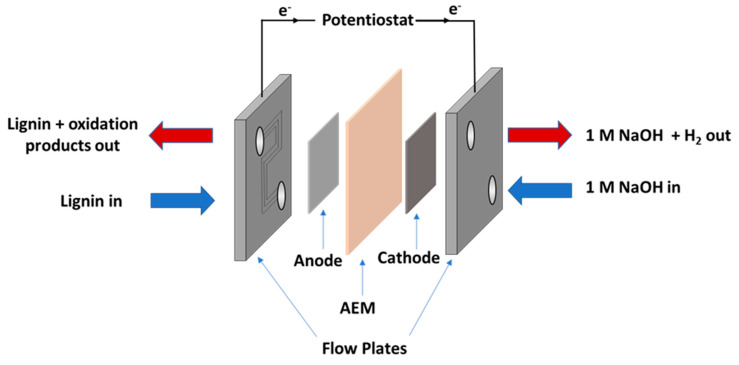
Diagram of the electrolyser cell setup used for lignin electrolysis. The electrolyser setup is a “zero-gap” configuration where the anode and cathode catalysts are sandwiched between the conductive flow plates. The potentiostat is connected to each flow plate by way of terminal spades with banana jacks, and so the flow plates also function as current collectors.

The flow cell was assembled as shown in Figure 1. The anolyte used was a solution of alkali lignin (10 g L^−1^ in 1 M NaOH) and the catholyte was an aqueous solution of NaOH (1 M). The feed solutions were transported to and from the cell using two Fisherbrand GP1100 general purpose peristaltic pumps at a flow rate of 10 mL min^−1^. The flow plates used were fabricated from stainless steel, with 6 channels (0.9 × 0.9 mm) through which the feedstock solutions were passed. Gaskets were cut from either 0.45 mm or 0.1 mm thick polytetrafluoroethylene (PTFE). The stack compression was 5.65 Nm and the active area of the membrane was 12.96 cm^2^. The temperature within the cell was controlled by heating the reservoirs of the feed solutions in an oil bath. The temperature was monitored using K-type thermocouples inserted into the inlet and outlet of the anodic side. Temperature data were recorded using a Pico TC-08 data logger and PicoLog software for Windows.

### 2.3. Electrochemical Characterisation of the Cell

Electrochemical impedance spectroscopy (EIS) and linear sweep voltammetry (LSV) data were recorded for the electrolyser cell using a Bio-Logic SP-150 potentiostat equipped with a VMP3B-20 20 A booster. Data were recorded and analysed using EC-Lab (v11.12). Unless stated otherwise, EIS was carried out under the following experimental parameters: electrolyte flow rate = 10 mL min^−1^, quiet time (resting at the DC bias potential) = 10 min, starting frequency = 1 MHz, ending frequency = 10 mHz, DC bias = 0.5 V (vs. the open circuit potential), AC excitation amplitude = 14.1 mV. LSV data were recorded at a scan rate of 0.5 mV s^−1^. The EIS data were fitted to an equivalent circuit, L_1_ + R_1_ + Q_1_/R_2_ using AfterMath (v1.5.9644, Pine Research Instrumentation Inc. (Durham, NC, USA)). The details of the components of the equivalent circuit are as follows: L_1_, which is an inductor; R_1_, which corresponds to the series resistance of the cell (R_s_); R_2_, which corresponds to the polarisation resistance of the cell (R_p_); Q_1_, which represents a constant phase element.

### 2.4. Preparation of PVIB Membrane

Firstly, poly (vinylidene fluoride-co-hexafluoropropylene) (PVDF-co-HFP, 10 g) was dissolved in dimethylacetamide (500 mL). Then, saturated NaOH in isopropanol (10 mL) was added dropwise, over 30 min, while the solution was stirred vigorously at room temperature. During this step the colourless solution turned light brown in colour. The resulting dehydrofluorinated PVDF-co-HFP was then precipitated in water, filtered and then rinsed 3–4 times with deionised water and dried under vacuum at 70 °C. This polymer was then dissolved in a round-bottomed flask containing dimethylacetamide, *N*-vinylimidazole (20% wt), and (vinylbenzyl)trimethylammonium chloride at different weight % concentrations (4%, 6%, 8% and 10%), giving four different types of PVIB membrane on the basis of the amount of (vinylbenzyl)trimethylammonium chloride added: PVIB-4, PVIB-6, PVIB-8 and PVIB-10, respectively. The copolymerisation reaction was initiated by addition of 0.1% of azobisisobutyronitrile (AIBN), and the mixture was continuously stirred at 60 °C for 8 h under an N_2_ atmosphere. The resulting viscous solution was cast onto a clean glass plate and dried under vacuum at 55 °C for 24 h. After this, the membrane was equilibrated in 1 M NaOH for 24 h to complete the exchange of Cl^−^ for OH^−^. These hydroxide-exchanged membranes were analysed after thoroughly washing with double distilled water 4–5 times.

### 2.5. Characterisation of the PVIB Membrane

A number of analytical techniques were used to characterise the structure, functional groups, surface and phase morphology of the as-prepared PVIB and its precursors. Functional group analysis of the samples was performed using a PerkinElmer FT-IR spectrometer. Surface and cross-sectional morphology on freshly prepared membranes was analysed on a field-emission electron microscope (FE-SEM) using a JEOL JEM 7100F (USA) instrument. TEM images were recorded with a JEOL JEM 2100 microscope. Scanning electron microscopy on membranes after use in the PVIB-based electrolyser was performed with a Philips XL30 ESEM instrument equipped with an Oxford Instruments Energy 250 energy dispersive spectrometer system at an acceleration voltage of 20 kV. Following use in a lignin-oxidising electrolyser, membranes were thoroughly washed and then submerged in ultrapure water for 2 h, before being oven-dried for 4 h at 55 °C. Samples cut from the membrane were then loaded onto 12 mm AGAR scientific conductive carbon tabs. Images were obtained with acceleration voltages between 12 kV and 20 kV.

To measure the ion-exchange capacity, 2.5 × 2.5 cm^2^ fragments of the prepared membrane were submerged in 1.0 N NaCl (AR grade) solution for 24 h in order for the membrane to be entirely converted to the form with the chloride counter ion. These membrane squares were then removed from solution, thoroughly washed with ultra-pure water and equilibrated in deionised water for 2 h to remove any excess chloride ions from the membrane surface. Finally, the membrane squares were dried in a vacuum oven for 4 h at 55 °C and then weighed. The chloride-saturated membrane was subsequently immersed in 0.1 M Na_2_SO_4_ in order to allow exchange of the chloride counter ions for SO_4_^2−^. The chloride ions thus released into solution were titrated by Mohr’s method using 0.001 N AgNO_3_ and dichromate solution as the indicator. The ion-exchange capacity (IEC) was then determined using the formula in Equation (1), where *V*_AgNO3_ is the volume of 0.001 N AgNO_3_ solution added and *M*_Dry_ is the dry mass of the membrane square:

(1) $$\mathrm{IEC}\;\left({\mathrm{meq}\;g}^{-1}\right)=\frac{0.001\;N\;\times V_{{\mathrm{AgNO}}_{3}}}{M_{\mathrm{Dry}}}\;$$

The ionic conductivity (σ) of the prepared membranes was measured at 30 °C using AC impedance spectroscopy with an AutoLab Model PGSTAT 30 potentiostat/galvanostat frequency response analyser. This instrument was connected to the conductivity cell. The conductivity cell itself was made in-house and consisted of two circular stainless-steel electrodes (each of effective area 1.0 cm^2^), each of which was encased in an acrylic outer cylinder (approximately 2 cm thick). The membrane was sandwiched between the two stainless-steel electrodes using 0.1 M NaCl as the conducting medium. The frequency of sinusoidal current perturbation was swept from 1 MHz to 1 Hz over the course of each experiment, and the current demanded was swept at 1 μA per second. The resulting Nyquist plot was then used to obtain the resistance of the membrane. The conductivity of the membrane was calculated by entering values for the membrane area (*A*), the distance between the electrodes (i.e., membrane thickness, *L*) and the resistance (*R*) into Equation (2) below:

(2) $$\sigma \;\left({S\;\mathrm{cm}}^{-1}\right)=\frac{L\;\left(\mathrm{cm}\right)}{R\;\left(\Omega \right)\times A\;\left({\mathrm{cm}}^{2}\right)}\;\;$$

The mechanical strength of the membrane samples (rectangular pieces of size 26 cm^2^) was studied by using a bursting strength tester machine (model No. 807DMP, Test Techno Consultants, Gujarat, India). The stability of the prepared PVIB membranes in alkaline media was studied by immersing the membranes in 5.0 M NaOH for 72 h at 30 °C. The mass and conductivity of the treated membranes were unaltered by this treatment, suggesting that they are stable in alkaline media at room temperature for at least 72 h.

## 3. Results

### 3.1. Properties of the PVIB Membrane

**Figure 2:**
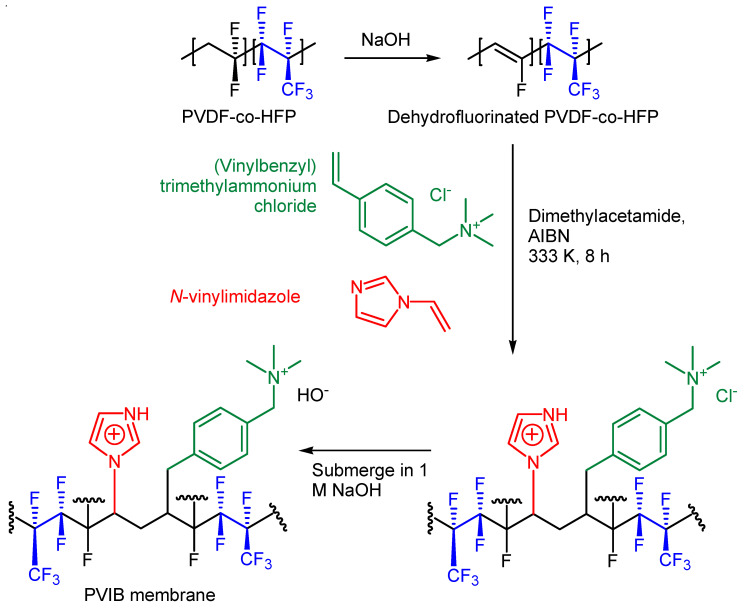
Synthesis and structure of the PVIB-based polymer.

A schematic of the synthetic route used to generate the PVIB polymer (according to the procedure in Section 2.4) is shown in Figure 2.

**Figure 3:**
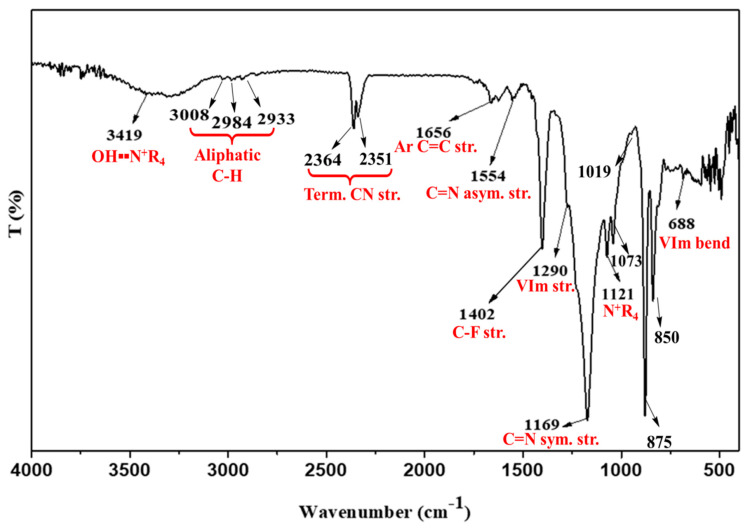
ATR-IR transmission spectrum of the PVIB anion-exchange membrane.

Co-polymerisation to produce PVIB was achieved via free radical polymerisation using *N*-vinylimidazole and (vinylbenzyl)trimethylammonium chloride monomers and AIBN as a radical initiator (see Figure 2). The transmission spectrum for PVIB was recorded between 4000 and 400 cm^−1^, as shown in Figure 3. The presence of absorption bands at 1402 cm^−1^ and 2933 cm^−1^, and 2984 cm^−1^ and 3008 cm^−1^ is attributed to the C-F stretching and methylene (-CH_2_-) stretching modes, respectively [38]. These peaks confirm the successful co-polymerisation reaction between the monomers and dehydrofluorinated PVDF-co-HFP.

Meanwhile, the peaks at 688 cm^−1^, 1073 cm^−1^ and 1290 cm^−1^ are assigned to the vinylimidazole bending and stretching modes, respectively, whilst the peaks at 1169 cm^−1^ and 1554 cm^−1^ are assigned to symmetric and anti-symmetric C=N stretches in the heterocyclic ring [39]. Finally, the peaks at 1121 cm^−1^, 1656 cm^−1^, 2352 cm^−1^, 2364 cm^−1^ and 3419 cm^−1^ confirm ionomer grafting: these peaks are attributed to the stretching modes of C-N^+^, aromatic C=C and O-H (bound water) associated with the quaternary ammonium groups [38,40]. Interestingly, the bands at 850 cm^−1^, 875 cm^−1^ and 1019 cm^−1^ additionally confirm the para-di-substitution and ring breathing of benzene, respectively [40]. No absorption bands at 960 or 1690 cm^−1^ (indicative of free vinyl groups) were observed, showing that addition across the double bond is complete [41].

**Figure 4:**
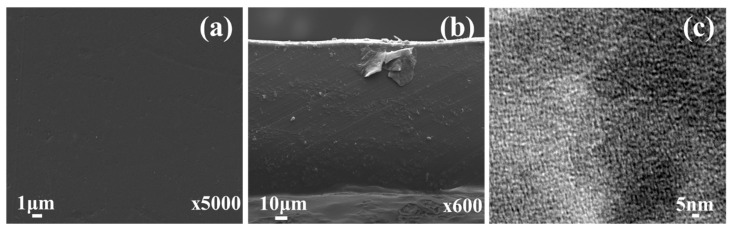
Microscopy images of the as-prepared PVIB-10 membrane: (**a**) SEM image of the membrane surface, (**b**) SEM image of the membrane cross-sectional morphology and (**c**) TEM image showing the nano-phase separated morphology of the PVIB-10 membrane.

Surface and cross-sectional images (Figure 4) were captured by FE-SEM at 15.4 kV incident beam energy. The results illustrate that these membranes (as-prepared) possess homogenous dense morphology (both surface and bulk) devoid of any cracks, pinholes, or any other deleterious morphology which might influence membrane performance (at least at the outset) during application.

Hydrophilic/hydrophobic nano-phase separation was examined using TEM. From Figure 4, it is apparent that dark and light regions can be distinguished on the nanometre (~3–5 nm) scale, and that the domains are well connected. The former is attributed to the sinuous (worm-like) hydrophilic domain for −N^+^(CH_3_)_3_ and the latter accounts for the hydrophobic fluorinated phase of PVDF-co-HFP [42]. Due to presence of both hydrophobic (PVDF-co-HFP) and hydrophilic (*N*-vinylimidazole) domains, the membrane forming material showed phase separation, which is responsible for the formation of the ion-conducting channels.

Table 1 shows the ion-exchange capacity (IEC) and hydroxide conductivity (*κ^m^*) for the series of PVIB membranes prepared in this work. The extent of ion migration through ion exchangers (in this case, the quaternary ammonium groups) is crucial for good membrane performance and depends upon the amount of charged functionality that is grafted into the polymer. The increase in IEC from 1.43 meq g^−1^ for PVIB-4 to 1.82 meq g^−1^ for PVIB-10 (a 27% increase) is attributed to the increasing degree of (vinylbenzyl) trimethylammonium grafting from 4 wt% to 10 wt% in the membrane matrix. The development of well-established ion percolating channels due to optimum swelling could be the underlying reason for this. A similar trend was observed for OH^−^ conduction through the membrane matrix: a~17% increase was recorded as this value ranged from 4.12 × 10^−2^ S cm^−1^ for PVIB-4 to 4.84 × 10^−2^ S cm^−1^ for PVIB-10. The membrane swelling ratio also increased with IEC, due to the associated improvement in the membranes’ hydrophilic nature. The mechanical stability of the prepared PVIB membranes was assessed by burst strength value (Table 1). Freshly-prepared PVIB-10 membrane gave a burst strength value of 8.93 kg cm^−2^, suggesting good mechanical stability for the as-prepared membrane.

### 3.2. Characterisation of the PVIB-Based Electrolyser

**Figure 5:**
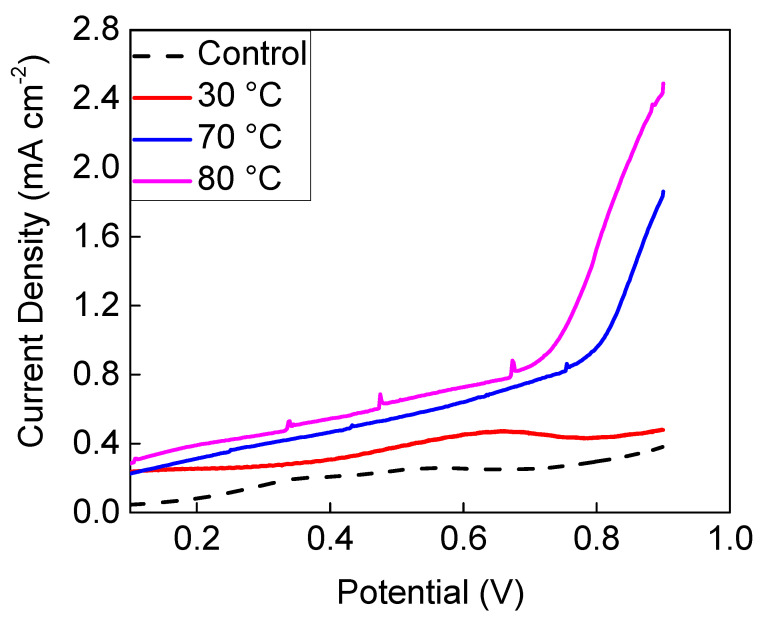
Polarisation curves recorded at 80 °C (pink trace), 70 °C (blue trace), and 30 °C (red trace) showing the behaviour of electrolysers using a commercial Fumapem membrane with an anolyte feed of 1 M NaOH containing 10 g L^−1^ alkali lignin, alongside a control for the electrolyser using only 1 M NaOH at room temperature as the anolyte (black-dashed trace).

To define a benchmark for the performance of our PVIB-based lignin electrolyser, we first constructed an electrochemical cell using the commercially-available anion-exchange membrane, Fumapem (FAA-3–50). The suitability of this membrane for use in a lignin electrolyser has previously been investigated by Caravaca et al. [30]. The first method of analysis used was linear sweep voltammetry (LSV). In this technique, the current is recorded as the cell potential is varied. Much like cyclic voltammetry, the recorded current is a function of the scan rate, with higher currents being recorded at the same potentials when using higher scan rates. We therefore selected a very low scan rate (0.5 mV s^−1^) in order to obtain a current density as close to the steady-state value as possible. Figure 5 shows a comparison of the performance of a cell using the components described in Section 2.1 and Section 2.2 and a Fumapem membrane at various temperatures using a catholyte feed of 1 M NaOH, and an anolyte feed of alkali lignin (10 g L^−1^) in 1 M NaOH. At 30 °C, the current density for lignin electrolysis was rather low and was similar to that achieved in a control without any lignin being present in the anolyte feed at room temperature (around 20 °C): compare the red solid and black-dashed traces in Figure 5. However, at the higher temperatures of 70 °C and 80 °C, significant increases in current density above the lignin-free background were evident, especially at cell potentials greater than 0.8 V. These results are broadly in agreement with those obtained by Caravaca et al. [30] with their analogous electrolyser and therefore show that the electrolyser configuration described in Section 2.1 and Section 2.2 is a valid setup in which to test the performance of the PVIB membrane for lignin electrolysis. We can, therefore, have some confidence in the comparisons that we shall draw between this PVIB membrane and commercially-available alternatives.

**Figure 6:**
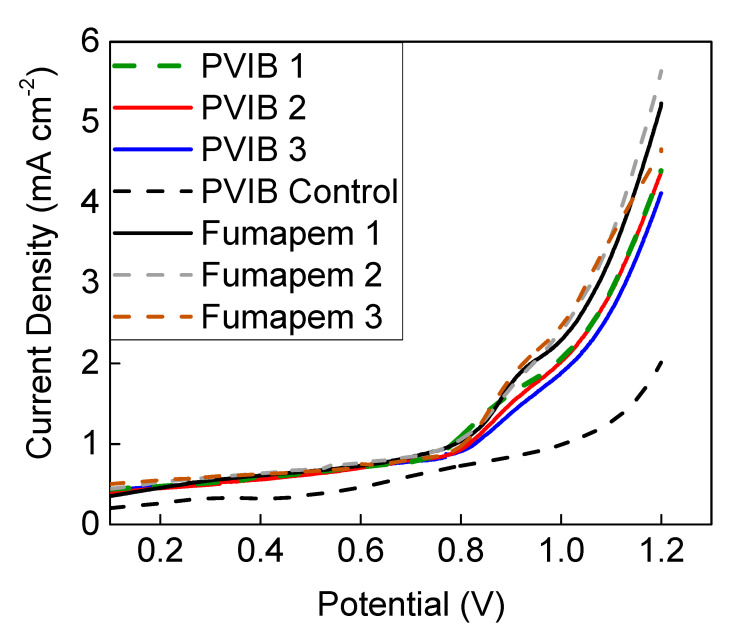
Polarisation curves at 70 °C showing the behaviour of electrolysers using commercial Fumapem and PVIB-10 with an anolyte feed of 1 M NaOH containing 10 g L^−1^ alkali lignin. The green-dashed line, solid red line and solid blue line show three repeat traces for the PVIB-10 membrane, and the black solid line, grey-dashed line and brown-dashed line show three repeat traces for the Fumapem membrane. A control for a PVIB-10-based system using only 1 M NaOH at 70 °C as the anolyte is also provided as the black-dashed trace.

LSV measurements at 70 °C were then repeated using this electrolyser setup, but having substituted the novel PVIB-10 membrane for Fumapem, as shown in Figure 6. Cell potentials were also scanned to more positive values in order to obtain higher current densities for lignin oxidation. A control measurement, shown in Figure 6 as the black-dashed trace, was also performed using PVIB-10 and 1 M NaOH as both the anolyte and catholyte (i.e., in the absence of lignin). The data in Figure 6 show that the PVIB-10-based electrolyser produced current densities almost identical to those produced by the Fumapem-based electrolyser across a range of cell potentials as high as 1.2 V. For example, at 0.9 V a current density of 1.5 mA cm^−2^ was achieved using PVIB-10 (vs. 1.9 mA cm^−2^ when using Fumapem) and at 1.2 V a current density of 4.4 mA cm^−2^ was achieved (vs. 5.3 mA cm^−2^ when using Fumapem). Three repeat runs for both PVIB-10 and Fumapem are shown in Figure 6, indicating that both membranes show fairly consistent performance in these swept-voltage experiments, with only a slight deterioration in performance for the PVIB-10 membrane evident in the third run.

**Figure 7:**
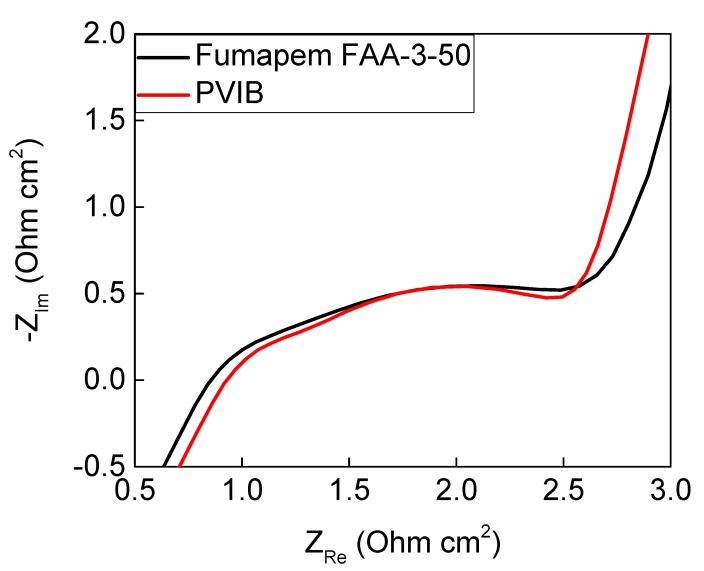
EIS comparison between commercial Fumapem and PVIB-10 at 70 °C when used in the electrolyser. Both the anolyte and catholyte were 1 M NaOH.

EIS was also performed at 70 °C on the PVIB-10 and Fumapem-based electrolysers in order to gain insights into the resistances that these membranes present for lignin oxidation. These data (Figure 7) show that the series resistance, R_s_, was found to be 0.87 Ω cm^2^ for the PVIB-10-based system versus 0.80 Ω cm^2^ for the Fumapem system, whilst the polarisation resistance, R_p_, for the PVIB-10 electrolyser was slightly lower than for the Fumapem system (1.47 Ω cm^2^ versus 1.59 Ω cm^2^). R_s_ can be obtained from Figure 7 by considering where the semi-circle first intercepts the *x*-axis at high frequency. In an ideal setting, R_p_ is then simply the difference between the high and low frequency intercepts of the *x*-axis. As the data in Figure 7 do not intercept the *x*-axis again at low frequency, R_p_ was obtained by fitting the data to an equivalent circuit as described in Section 2.3. The polarisation resistance can be thought of as the sum of the resistances associated with polarising the cell, such as the energy barriers associated with the HER and OER, and kinetic and mass transfer effects. From the technical datasheet for Fumapem provided by the manufacturer, the stated OH^−^ conductivity for this membrane is lower than the observed OH^−^ conductivity of PVIB-10 (4.0−4.5 × 10^−2^ S cm^−1^ for Fumapem versus 4.84 × 10^−2^ S cm^−1^ for PVIB-10). This slightly better in conductivity for (freshly-prepared) PVIB-10 versus Fumapem is borne out by the lower value of R_p_. A comparison between the ion-exchange capacity and hydroxide conductivity of PVIB-10 and a selection of anion-exchange membranes from the recent literature is given in Table 2. Taken together, the EIS and LSV data suggest that PVIB and Fumapem show very similar underlying performance when employed as anion-exchange membranes in lignin-oxidising electrolysers, at least on the short timescales of the LSV experiments.

**Figure 8:**
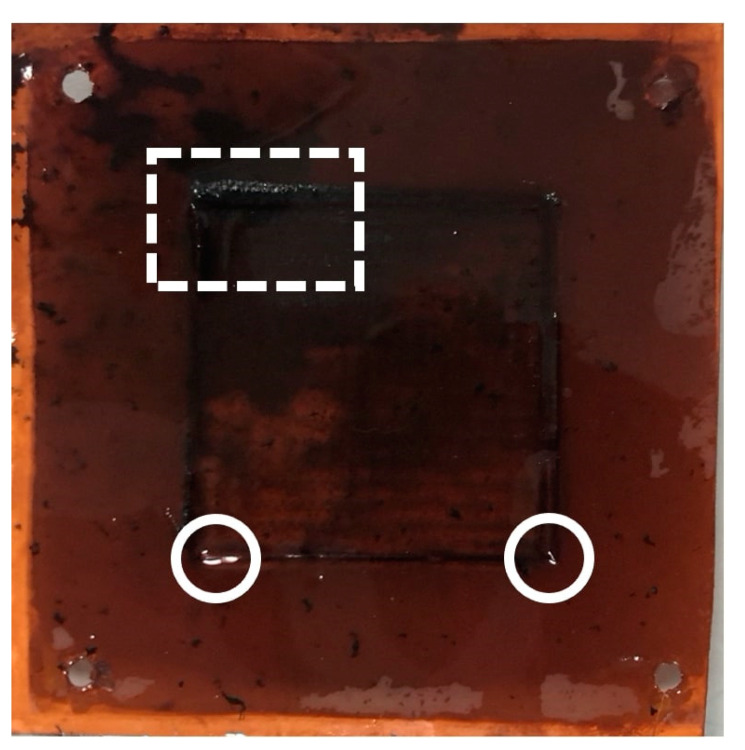
Warped and holed PVIB-10 membrane after repeated use. The total area of the membrane is a square of dimensions 7 cm × 7 cm.

**Figure 9:**
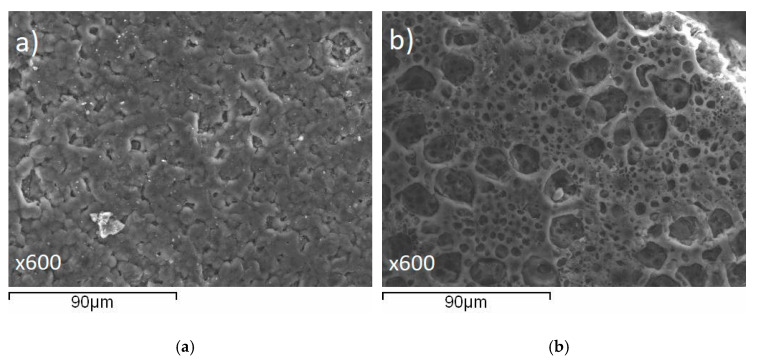
Microscopy images (at ×600 magnification) of the PVIB-10 membrane after testing in a lignin-oxidising electrolyser: (**a**) SEM image of an area of the membrane surface that was apparently undamaged to the naked eye and (**b**) SEM image of an area of the membrane surface where damage was already evident by eye.

Upon repeated testing of the PVIB-10 membranes in this electrolyser setup, it was noted that resistances increased and current densities decreased at any given cell potential, suggesting that the membranes were unstable under extended use for lignin oxidation. Examination of the membranes after such repeated testing also showed that they had become less mechanically robust and more prone to warping and holing over the course of these experiments. Figure 8 shows typical examples of such damage, which tended to manifest most obviously around the edges of the active area (visible as the black square in Figure 8) where the membrane was most warped. Such warping is especially evident at the top left of the active area (highlighted with the-dashed rectangle) whilst holes are present in the bottom left and right of Figure 8 (circled). The morphology of the membranes after use in the PVIB-based electrolyser was examined by SEM, as shown in Figure 9. Panel (a) in Figure 9 shows an image of an area of the membrane that appeared relatively undamaged to the naked eye. Nevertheless, considerable cracking is evident, and the morphology is now much less uniform when compared with pristine samples (see Figure 4). Meanwhile, Figure 9b shows an image of an area where damage was already apparent by eye, and in this case extensive holing of the membrane on the microscale is evident. Clearly, then, the membrane has suffered significant deterioration as a result of use in the electrolyser. Further insight into the higher resistance of the membrane after use was found by considering the ion-exchange capacity of the used membranes: after repeated use, the ion-exchange capacity (as measured by the protocol in Section 2.5) was found to decrease to around 0.4 meq g^−1^ for PVIB-10 (compared to 1.82 meq g^−1^ for pristine membranes, see Table 1). Such a decrease in ion-exchange capacity suggests chemical degradation of the membrane by removal of cationic groups. Submersion in alkaline medium alone does not reduce the ion-exchange capacity of the membranes at room temperature (see Section 2.5), and so the cause of the membrane degradation is most likely a combination of applied potential during membrane testing, together with the elevated temperature and basic medium.

**Figure 10:**
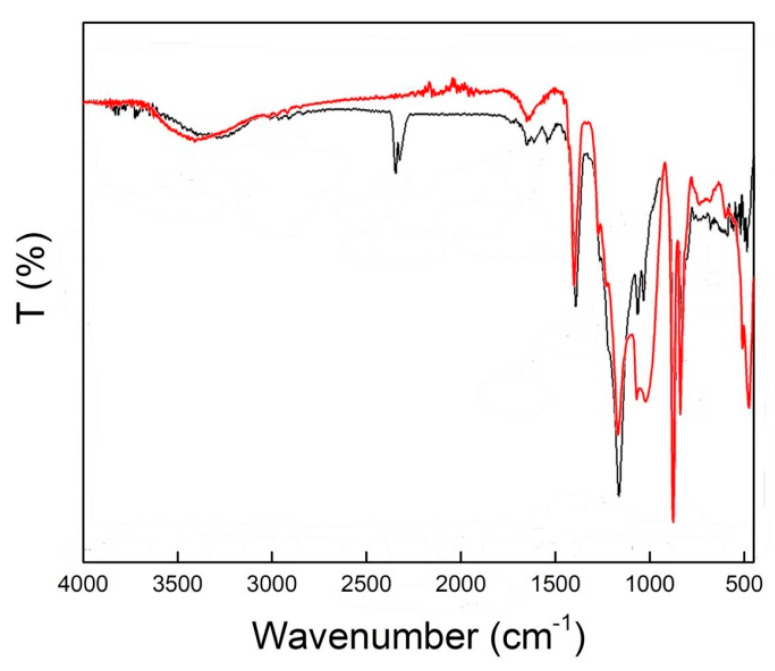
ATR-IR transmission spectrum of the PVIB-10 anion-exchange membrane before use (black) and after extensive use (red).

In terms of the mechanism of membrane degradation that leads to this loss of ion-exchange capacity, Sata et al. have previously shown [43] that anion-exchange membranes bearing benzyl trimethylammonium groups can suffer chemical degradation after immersion in highly alkaline solutions at temperatures of 75 °C (very similar conditions to those that we use here). These authors attributed their degradation to decomposition of the benzyl trimethylammonium moieties via attack of hydroxide at the carbon in between the benzene ring and the trimethylammonium unit through an S_N_2 mechanism, yielding free trimethylamine and the benzyl alcohol derivative of the polymer. Moreover, there is evidence that this mechanism is also operating in our case: an IR spectrum collected on used membranes (Figure 10, red trace) shows that the peaks at 2352 cm^−1^, 2364 cm^−1^ (assigned to the terminal C-N^+^ stretches in the quaternary amine groups in Figure 3) are completely absent in the used membranes, suggesting that these groups have been mostly cleaved during operation.

**Figure 11:**
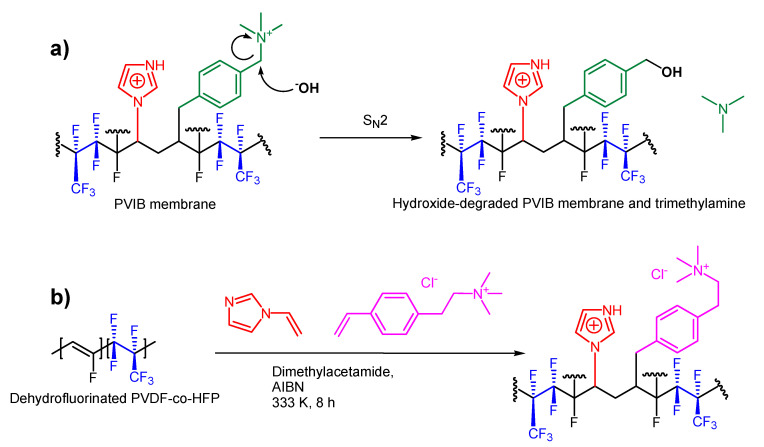
(**a**) A possible hydroxide-meditated route to membrane degradation involving the cleavage of trimethylamine from the polymer. (**b**) A potential strategy for the synthesis of a more stable analogue of PVIB: The vinylbenzene derivative highlighted in pink replaces (vinylbenzyl) trimethylammonium chloride in the general synthetic scheme shown in Figure 2, with the extra methylene group disfavouring the facile S_N_2 mechanism shown in panel (**a**).

If cleavage of the trimethylamine groups is indeed operating in our case, then inserting an additional methylene unit(s) in between the trimethylammonium moiety and the aromatic ring would be expected to significantly retard this S_N_2 mechanism and hence lead to increased stability of the membrane in highly alkaline solution. A similar strategy has been shown to be effective in previous work reported in the literature [44,45,46]. The synthesis of a suitable monomer for this purpose that could be used in a synthetic scheme similar to that shown in Figure 2 has been reported [47]. Figure 11a shows the putative hydroxide-mediated membrane degradation mechanism and Figure 11b shows a structure for the proposed more robust polymer using this alternative monomer that might show slower degradation in alkaline solution at elevated temperature.

Regardless of the cause of this membrane degradation, such behaviour has so far prevented us from obtaining reliable data for steady-state operation (including current-time curves, hydrogen yields and investigations of the products of lignin electrolysis) to compare with that for a Fumapem-based electrolyser. Work to improve the longevity of these membranes so that such data can be obtained is currently underway in our laboratories.

## 4. Conclusions

In summary, we have described the synthesis and characterisation of a novel anion-exchange membrane, PVIB. This membrane was then employed in a continuous flow lignin oxidation cell, using Pt/C and Pt/Ru catalysts impregnated on carbon cloth. It was found that the performance of the novel membrane was competitive with a commercial equivalent, Fumapem, over short timescales, although long-term durability remains an ongoing challenge. Taken together, these results give us some encouragement that this (or a similar) material could one day be developed into a cost-effective conductive separator for electrolysers that simultaneously oxidise lignin and generate hydrogen.

## Figures and Tables

**Table 1:** Physicochemical, electrochemical and mechanical parameters for the series of PVIB anion-exchange membranes prepared in this work. The codes applied to the different membranes indicate the weight % of (vinylbenzyl) trimethylammonium chloride added during synthesis, as mentioned in the text.

Membrane	IEC (meq g^−1^)	*κ^m^* × 10^−2^ (S cm^−1^)	Swelling Ratio (%)	Burst Strength (kg cm^−2^)
PVIB-4	1.43	4.12	13.2	8.05
PVIB-6	1.62	4.27	15.9	8.34
PVIB-8	1.77	4.49	17.9	8.57
PVIB-10	1.82	4.84	20.5	8.93

**Table 2:** Ion-exchange capacity (IEC) and hydroxide conductivity (*κ^m^*) values for PVIB, and a selection of anion-exchange membranes from papers referenced in this work.

Membrane	IEC (meq g^−1^)	*κ^m^* × 10^−2^ (S cm^−1^)	Ref
PVIB-10	1.82 ^a^	4.84	This work
GT82-5	3.84 ^a^ (3.76 ^b^)	10.9	[14]
GT64-15	3.26 ^a^ (3.28 ^b^)	6.2	[14,15]
XL4-PNB-X_34_-Y_66_	3.43 ^b^	8.68	[16]
Fumapem FAA-3-50	2.02	4.0–4.5	[30]

^a^ IEC determined by titration; ^b^ IEC determined by ^1^H NMR.

## Data Availability

All data generated or analysed during this study are included in this published article. The data which underpin this work are available at http://dx.doi.org/10.5525/gla.researchdata.1153 (accessed on 1 June 2021).

## References

[1] Dincer I. (1999). Environmental impacts of energy. Energy Policy.

[2] Roger I., Shipman M.A., Symes M.D. (2017). Earth-abundant catalysts for electrochemical and photoelectrochemical water splitting. Nat. Rev. Chem..

[3] Lewis N.S., Nocera D.G. (2006). Powering the Planet: Chemical Challenges in Solar Energy Utilization. Proc. Natl. Acad. Sci. USA.

[4] Wallace J.S., Ward C.A. (1983). Hydrogen as a Fuel. Int. J. Hydrogen Energy.

[5] Carmo M., Fritz D.L., Mergel J., Stolten D. (2013). A comprehensive review on PEM water electrolysis. Int. J. Hydrogen Energy.

[6] Gahleitner G. (2013). Hydrog. From renewable electricity: An international review of power-to-gas pilot plants for stationary applications. Int. J. Hydrogen Energy.

[7] Sommer E.M., Martins L.S., Vargas J.V.C., Gardolinski J.E.F.C., Ordonez J.C., Marino C.E.B. (2012). Alkaline membrane fuel cell (AMFC) modelling and experimental validation. J. Power Sources.

[8] Chi J., Yu H. (2018). Water electrolysis based on renewable energy for Hydrog. production. Chin. J. Catal..

[9] Millet P., Andolfatto F., Durand R. (1996). Design and Performance of a Solid Polymer Electrolyte Water Electrolyzer. Int. J. Hydrogen Energy.

[10] Grigoriev S., Baranov I., Millet P., Li Z., Fateev V. (2009). Optimization of porous current collectors for PEM water electrolysers. Int. J. Hydrogen Energy.

[11] Schalenbach M., Carmo M., Fritz D.L., Mergel J., Stolten D. (2013). Pressurized PEM water electrolysis: Efficiency and gas crossover. Int. J. Hydrogen Energy.

[12] Vincent I., Kruger A., Bessarabov D. (2017). Development of efficient membrane electrode assembly for low cost Hydrog. production by anion exchange membrane electrolysis. Int. J. Hydrogen Energy.

[13] Varcoe J.R., Atanassov P., Dekel D.R., Herring A.M., Hickner M.A., Kohl P.A., Kucernak A.R., Mustain W.E., Nijmeijer K., Scott K. (2014). Anion-exchange membranes in electrochemical energy systems. Energy Environ. Sci..

[14] Mandal M., Huang G., Hassan N.U., Peng X., Gu T., Brooks-Starks A.H., Bahar B., Mustain W.E., Kohl P.A. (2000). The Importance of Water Transport in High Conductivity and High-Power Alkaline Fuel Cells. J. Electrochem. Soc..

[15] Hassan N.U., Mandal M., Huang G., Firouzjaie H.A., Kohl P.A., Mustain W.E. (2020). Achieving High-Performance and 2000 h Stability in Anion Exchange Membrane Fuel Cells by Manipulating Ionomer Properties and Electrode Optimization. Adv. Energy Mater..

[16] Mandal M., Huang G., Kohl P.A. (2019). Highly Conductive Anion-Exchange Membranes Based on Cross-Linked Poly(norbornene): Vinyl Addition Polymerization. ACS Appl. Energy Mater..

[17] Zhang C., Zhang W., Wang Y. (2020). Diffusion Dialysis for Acid Recovery from Acidic Waste Solutions: Anion Exchange Membranes and Technology Integration. Membranes.

[18] Xiang C., Padapantonakis K.M., Lewis N.S. (2016). Principles and implementations of electrolysis systems for water splitting. Mater. Horiz..

[19] Lalvani S.B., Rajagopal P. (1992). Lignin-Augmented Water Electrolysis. J. Electrochem. Soc..

[20] Ju H.K., Badwal S., Giddey S. (2018). A comprehensive review of carbon and hydrocarbon assisted water electrolysis for Hydrog. production. Appl. Energy.

[21] Pushkareva I.V., Pushkarev A.S., Grigoriev S.A., Lyutikova E.K., Akel’kina S.V., Osina M.A., Slavcheva E.P., Fateev V.N. (2016). Electrochemical Conversion of Aqueous Ethanol Solution in an Electrolyzer with a Solid Polymer Electrolyte. Russ. J. Appl. Chem..

[22] Caravaca A., De Lucas-Consuegra A., Calcerrada A.B., Lobato J., Valverde J.L., Dorado F. (2013). From biomass to pure hydrogen: Electrochemical reforming of bio-ethanol in a PEM electrolyser. Appl. Catal. B Environ..

[23] Sasikumar G., Muthumeenal A., Pethaiah S.S., Nachiappan N., Balaji R. (2008). Aqueous methanol electrolysis using proton conducting membrane for Hydrog. production. Int. J. Hydrogen Energy.

[24] McHugh P.J., Stergiou A.D., Symes M.D. (2020). Decoupled Electrochemical Water Splitting: From Fundamentals to Applications. Adv. Energy Mater..

[25] Movil-Cabrera O., Rodriguez-Silva A., Arroyo-Torres C., Staser J.A. (2016). Electrochemical conversion of lignin to useful chemicals. Biomass Bioenergy.

[26] Ghatak H.R. (2006). Electrolysis of black liquor for Hydrog. production: Some initial findings. Int. J. Hydrogen Energy.

[27] Ghatak H.R., Kumar S., Kundu P.P. (2008). Electrode processes in black liquor electrolysis and their significance for Hydrog. production. Int. J. Hydrogen Energy.

[28] Lalvani S.B., Rajagopal R. (1993). Hydrog. Production from Lignin-Water Solution by Electrolysis. Holzforschung.

[29] Bateni F., NaderiNasrabadi M., Ghahremani R., Staser J.A. (2019). Low-Cost Nanostructured Electrocatalysts for Hydrog. Evolution in an Anion Exchange Membrane Lignin Electrolysis Cell. J. Electrochem. Soc..

[30] Caravaca A., Garcia-Lorefice W.E., Gil S., De Lucas-Consuegra A., Vernoux P. (2019). Towards a sustainable technology for H_2_ production: Direct lignin electrolysis in a continuous-flow Polymer Electrolyte Membrane reactor. Electrochem. Commun..

[31] Tolba R., Tian M., Wen J., Jiang Z.H., Chen A. (2010). Electrochemical oxidation of lignin at IrO_2_-based oxide electrodes. J. Electroanal. Chem..

[32] Liu W., Cui Y., Du X., Zhang Z., Chao Z., Deng Y. (2016). High efficiency Hydrog. evolution from native biomass electrolysis. Energy Environ. Sci..

[33] Du X., Liu W., Zhang Z., Mulyadi A., Brittain A., Gong J., Deng Y. (2017). Low-energy catalytic electrolysis for simultaneous Hydrog. evolution and lignin depolymerization. ChemSusChem.

[34] Hibino T., Kobayashi K., Nagao M., Teranishi S. (2017). Hydrog. Production by Direct Lignin Electrolysis at Intermediate Temperatures. ChemElectroChem.

[35] NaderiNasrabadi M., Bateni F., Chen Z., Harrington P.B., Staser J.A. (2019). Biomass-Depolarized Electrolysis. J. Electrochem. Soc..

[36] Movil O., Garlock M., Staser J.A. (2015). Non-precious metal nanoparticle electrocatalysts for electrochemical modification of lignin for low-energy and cost-effective production of hydrogen. Int. J. Hydrogen Energy.

[37] Tsehaye M.T., Alloin F., Iojoiu C. (2019). Prospects for Anion-Exchange Membranes in Alkali Metal–Air Batteries. Energies.

[38] Sharma P.P., Yadav V., Rajput A., Kulshrestha V. (2018). PVDF-g-poly (styrene-co-vinylbenzyl chloride) based anion exchange membrane: High salt removal efficiency and stability. Desalination.

[39] Kuba A.G., Smolin Y.Y., Soroush M., Lau K.K.S. (2016). Synthesis and integration of poly (1-vinylimidazole) polymer electrolyte in dye sensitized solar cells by initiated chemical vapor deposition. Chem. Eng. Sci..

[40] Sharma J., Misra S.K., Kulshrestha V. (2021). Internally Cross-linked Poly (2, 6-dimethyl-1, 4-phenylene ether) based Anion Exchange Membrane for Recovery of different Acids by Diffusion Dialysis. Chem. Eng. J..

[41] Lebedeva O.V., Pozhidaev Y.N., Malakhova E.A., Raskulova T.V., Chesnokova A.N., Kulshrestha V., Pozdnyakov A.S. (2020). Sodium p-Styrene Sulfonate–1-Vinylimidazole Copolymers for Acid–Base Proton-Exchange Membranes. Membr. Membr. Technol..

[42] Wang X.Q., Lin C.X., Zhang Q.G., Zhu A.M., Liu Q.L. (2017). Anion exchange membranes from hydroxyl-bearing poly(ether sulfone)s with flexible spacers via ring-opening grafting for fuel cells. Int. J. Hydrogen Energy.

[43] Sata T., Tsujimoto M., Yamaguchi T., Matsusaki K. (1996). Change of anion exchange membranes in an aqueous sodium hydroxide solution at high temperature. J. Membr. Sci..

[44] Mandal M., Huang G., Kohl P.A. (2019). Anionic multiblock copolymer membrane based on vinyl addition polymerization of norbornenes: Applications in anion-exchange membrane fuel cells. J. Membr. Sci..

[45] Jeon J.Y., Park S., Han J., Maurya S., Mohanty A.D., Tian D., Saikia N., Hickner M.A., Ryu C.Y., Tuckerman M.E. (2019). Synthesis of Aromatic Anion Exchange Membranes by Friedel–Crafts Bromoalkylation and Cross-Linking of Polystyrene Block Copolymers. Macromolecules.

[46] Mandal M., Huang G., Hassan N.U., Mustain W.E., Kohl P.A. (2020). Poly(norbornene) anion conductive membranes: Homopolymer, block copolymer and random copolymer properties and performance. J. Mater. Chem. A.

[47] Wakabayashi K., Shimamura M., Akashi Y., Otake S., Matsuda T., Ito M., Noguchi A., Mori H. (2013). Developer Carrier and Developing Device.

